# Reviewers: the soul of a scientific journal

**DOI:** 10.1590/S1677-5538.IBJU.2017.02.01

**Published:** 2017

**Authors:** Sidney Glina

**Affiliations:** MD, PhD. Professor Titular Disciplina de Urologia da Faculdade de Medicina do ABC, Santo André, SP, Brasil

Peer review system is like democracy: it is absolutely imperfect, but the best available. It allows criticism to articles, suggestions for improvement under different perspectives and allows the editor to make a fairer decision about the future of an article.

The reviewer is the soul of a medical journal like International Brazilian Journal of Urology. He/she dictates the quality of publishing, the velocity by which each article reaches the reader, being a voluntary job that takes time. A good reviewer is rapidly identified by different magazines and is overloaded by many requests.

A good reviewer is not necessarily famous in his/hers specialty, but a committed person that spends precious time without payment in order to evaluate a paper and to decide if its valuable enough to be published or if it needs improvements and guidance to authors.

International Brazilian Journal of Urology has received 641 new submissions in 2016, added to other 275 that were revised and resubmitted. 456 reviewers participated in that task and it is important to nominate, as we have been doing in the past years, the most efficient and more collaborative.

We created a formula by which this choice is not subjective and only based on the opinion of the Associated Editors:

***Reviewer score****= (completed revisions/invitations)x R-score***

***Median time consumed for revision***

***The result is multiplied by 100.***

***R-score: given note that each AE gives to each revision (1= bad revision to 3-excelent review)***

Based on that formula we have elected the 5 most efficient reviewers of 2016 and we specially thanks them for their dedication:

John Denstedt, University of Western Ontario London, ON, Canada

Fabio Vicentini, Departamento de Urologia, Hospital das Clínicas, Faculdade de Medicina da Universidade de São Paulo, SP, Brasil

Renato Pedro, UNICAMP, Campinas, SP, Brasil and Hospital Israelita Albert Einstein, São Paulo, SP, Brasil

Marco Arap, Hospital Sírio Libanês, São Paulo, SP, Brasil

Lisias Castilho, Instituto de Ensino e Pesquisa do Hospital Sírio Libanês, São Paulo, SP, Brasil

Al of them evaluated at least 10 papers in 2016 and took 2 to 6 days to perform the revision. This effort and the agility of the Associated Editors allowed a median time of 54.2 days from submission to final decision for publishing, with an acceptance rate of 27.1%.

With the update of the journal website (www.intbrazjurol.com.br) and the policy of fast publication on line (Ahead of Print) of the accepted papers, today it is possible to have a paper published at International Brazilian Journal of Urology and consequently at Medline in a median time of 90 days after submission.

Another novelty of the website (very important to Brazilian authors and urologists) is the possibility to access on line all editions in full of the old Jornal Brasileiro de Urologia, since number 1, 1974.

The International Brazilian Journal of Urology is totally costed and supported by Brazilian Society of Urology and all publishing production is made by regularly employed officials at the Society headquarters. The joint effort of Ricardo de Morais, Technical Editor, Bruno Nogueira, Production Editor and Patricia Gomes, Secretary, has made our Journal more interactive and more competitive over the years.

Sidney Glina, MD, PhD Professor Titular Disciplina de Urologia da Faculdade de Medicina do ABC, Santo André, SP, Brasil

